# Dynamics of resilience in forced migration: a 1-year follow-up study of longitudinal associations with mental health in a conflict-affected, ethnic Muslim population

**DOI:** 10.1136/bmjopen-2014-006000

**Published:** 2015-02-16

**Authors:** Chesmal Siriwardhana, Melanie Abas, Sisira Siribaddana, Athula Sumathipala, Robert Stewart

**Affiliations:** 1 King's College London (Institute of Psychiatry), London, UK; 2 Institute for Research & Development, Colombo, Sri Lanka; 3 Faculty of Medical Science, Anglia Ruskin University, UK

**Keywords:** MENTAL HEALTH, PUBLIC HEALTH, STATISTICS & RESEARCH METHODS

## Abstract

**Objective:**

The concept of ‘resilience’ is of increasing interest in studies of mental health in populations facing adversity. However, lack of longitudinal data on the dynamics of resilience and non-usage of resilience-specific measurements have prevented a better understanding of resilience-mental health interactions. Hence, the present study was conducted to investigate the stability of levels of resilience and its associations with sociodemographic and mental health exposures in a conflict-affected internal-migrant population in Sri Lanka.

**Design:**

A prospective follow-up study of 1 year.

**Setting:**

Puttalam district of North Western province in postconflict Sri Lanka (baseline in 2011, follow-up in 2012).

**Participants:**

An ethnic Muslim population internally displaced 20 years ago (in 1990) from Northern Sri Lanka, aged 18 or above and currently in the process of return migration.

**Measures:**

It was hypothesised that levels of resilience would be associated with mental health outcomes. Resilience was measured on both occasions using the 14-item Resilience Scale (RS-14), social support by the Multidimensional Social Support Scale and Lubben Social Network Scale and common mental disorders by the Patient Health Questionnaire (PHQ).

**Results:**

Of 450 participants interviewed at baseline in 2011, 338 (75.1%) were re-interviewed in 2012 after a 1-year follow-up. The mean resilience scores measured by RS-14 were 80.2 (95% CI 78.6 to 81.9) at baseline and 84.9 (83.5 to 86.3) at follow-up. At both time points, lower resilience was independently associated with food insecurity, lower social support availability and social isolation. At both time points, there were significant associations with common mental disorders (CMDs) in unadjusted analyses, but they only showed independence at baseline. The CMD prevalence, maintenance and incidence at follow-up was 8.3%, 28.2% and 2.2%, respectively.

**Conclusions:**

In this displaced population facing a potential reduction in adversity, resilience was more strongly and robustly associated with economic and social factors than with the presence of mental disorder.

**Strengths and limitations of this study**

- To our knowledge, this study is the first of its kind to measure resilience longitudinally by using a specific tool among a population of adult forced internal migrants affected by conflict-driven prolonged displacement globally. A number of important demographic, economic and social variables are explored consistently across two time points, coinciding with the assumed reduction in adversity during postconflict and return-migration periods.
- Strengths of the study include complete participation at baseline and a reasonable follow-up rate (in the context of the complex nature of factors affecting the follow-up of study population in the postconflict milieu), maximising generalisability and reducing likelihood of bias. It also addresses key gaps in the field of resilience research by being longitudinal in design and by looking at associations between resilience levels and mental health outcomes.
- Limitations include the contextual appropriateness of the resilience measuring instrument, differential attrition and selection of time points. Cultural validity of the instrument and cultural variations or specificity of resilience may have affected the results. Capturing of only two time points may have limited the amount of information. Sample size limitations may have affected the analyses.

## Introduction

For several decades, resilience has received attention as a construct of psychological resistance against adversity.^1^ Advanced mainly through work in developmental psychopathology, the construct of resilience has been explored in biological, psychosocial, genetic and neurobiological fields.^2^ ^3^ Resilience has been studied in the backdrop of protective and risk factors related to positive or negative psychological outcomes, and also on individual, social and ecological levels.^4^ The definition and conceptualisation of resilience have received extensive attention, although limited by inherent construct issues and non-uniformity, especially in mental health.^3^ ^4^

In general, resilience has been defined and described as an ability in children and adults to adapt to, adjust to or overcome chronic or acute adversity, providing protection against the development of psychopathology.^1^ ^5^ However, Bonanno^6^ criticises resilience that is considered as a personality characteristic, linked to an absence of pathology and/or assumed to be an indicator of good health. Instead, he advocates an approach that involves clear temporal delineation of an adverse event, recognising resilience as a stable adjustment and measurement of resilience at multiple time points postadversity.^6^

Conflict-driven forced displacement has increased over the years, which is directly linked to increased burden of mental disorders in affected populations.^7^ Both externally (cross-border) and internally (within-country) displaced forced migrants have increased prevalence of depression, post-traumatic stress disorder (PTSD), anxiety, somatisation and other mental disorders.^7^ However, resilience (individual as well as communal) may act as a potential protective and mediating factor.^8–10^ Low resilience associated with prolonged displacement, on-going adversity and older age of forced migrants was found to predict poor mental health outcomes, while higher levels of resilience associated with better socioeconomic conditions, younger migration age and social support were associated with better outcomes.^8^ ^10–14^ Longitudinal trajectories of resilience outcomes in conflict-affected populations can be useful to gain a better understanding of these associations. However, longitudinal data on the dynamics of resilience in conflict-affected populations are scarce.^8^ ^10^

This paper presents findings from a 1-year follow-up study conducted among a population of internally displaced forced migrants from Sri Lanka, affected by over 20 years of prolonged displacement.^15–17^ This population has received very little attention about the mental health impact of prolonged displacement and the role of resilience in the long term.^15^ The study aimed to explore recent changes in resilience in this conflict-affected population, and associations with mental health outcomes in relation to a range of social, economic and demographic variables. It was envisaged that the study will provide longitudinal data on resilience outcome trajectories, which is inadequately available in the current literature.

## Methodology

### Study setting, design and participants

The study was carried out among a group of ethnic Muslims who had been displaced due to conflict from the Northern Province of Sri Lanka in 1990 and had been living since then in displacement in the North Western Province.^16^ ^17^ The study focused on this particular group of displaced ethnic Muslims as they had received little attention on their mental health status despite the prolonged displacement status.^15^ The study consisted of two components: (1) a base-line cross-sectional study and (2) a follow-up study. The baseline *COmmon Mental Disorders and Resilience Among Internally Displaced in Sri Lanka (COMRAID)* study was conducted in 2011, 2 years after the end of conflict in Sri Lanka and the follow-up phase was conducted in 2012, after a 1-year interval.^15^ The baseline cross-sectional study was conducted to assess the prevalence of common mental disorders (CMDs) and resilience in the specific population and the same sample was followed up for a period of 1 year to assess the same variables.^15^ Incidence of CMDs among those who were free at the baseline was also determined.

Although the conflict had ended before the baseline study started, return migration had barely started due to landmine clearing operations and lack of basic infrastructure in the areas of origin. However, return migration had picked up pace by the follow-up phase. The source population, including their forced migration history, has been previously described.^15–17^ COMRAID baseline included a sample of 450 adult participants using a multistage random sampling strategy that incorporated the Kish method at household level to recruit participants (one per household) from displacement camps and relocation villages.^15^ The sampling strategy was designed to account for forced migrants living in resettlement camps and also those in more permanent settlements. Population proportions were considered in the sampling calculations. The sample size and sampling strategy have been described separately in a previous publication.^15^ Participants were aged 18 or above, of Sri Lankan nationality, with a history of conflict-driven migration from Mannar district of Northern Province (or born to at least one parent with a similar migration background) and residing in the Kalpitiya administrative division area of Puttalam district in the North Western province.^15^ All sampled residents agreed to take part. The follow-up phase (COMRAID-R) included 338 (75%) of the baseline participants successfully traced and re-interviewed. The study methodology has been previously described.^15^

### Measurements

Near-identical measurements were obtained in both study phases through a structured interview conducted by trained research assistants (a team of 7) using Tamil language versions (the language spoken by the ethnic Muslims in Sri Lanka). Each of the instruments included in the interview was translated and back translated, subsequently checked for semantic and cultural validity, and the full questionnaire was piloted prior to field usage.^15^

Resilience was measured using the 14-item version of the Resilience Scale (RS 14).^18^ The original 25-item Resilience Scale (RS-25) was the first instrument to directly measure resilience.^19^ It is built on five underlying characteristics of resilience (a purposeful life, perseverance, equanimity, self-reliance and existential aloneness) that are collectively termed as the ‘resilience core’.^19–21^ The shorter RS-14 version was developed later, containing items that are measuring the same resilience core characteristics.^18^ ^21^ Each item of the RS-14 is scored on a seven-point Likert scale with respondent choices ranging from 1 (strongly disagree) to 7 (strongly agree). Total scores are conventionally grouped into six subcategories: very low (14–56 points), low (57–64), on low-end (65–73), moderate (74–81), moderately high (82–90), high (91–98). RS-25 and RS-14 have both been used in a wide variety of clinical and community populations to measure resilience including traumatised migrants.^18^ Both are available in several languages.^18^ The RS-14 is relatively quick to administer (4–5 min), inexpensive and simple to analyse.^21^ The internal consistency of the original English version of RS-14 is high, with a Cronbach α score of 0.93.^21^ RS-14 was not previously used or validated in Sri Lanka. The α coefficients of Tamil language versions used in the baseline and follow-up phases were 0.65 and 0.97, respectively. An exploratory factor analysis was carried out on the Resilience Scale for the two data sets and robust single solutions were observed at both instances (eigenvalue at baseline—6.80, eigenvalue at follow-up—9.96), in line with the factor analysis findings from the original RS-14 (eigenvalue—7.29).^21^

In both phases, CMD (depression, anxiety and somatoform disorder) was measured using the Primary Care Evaluation of Mental Disorders—Patient Health Questionnaire (PRIME MD PHQ) and PTSD was ascertained using Section-K of the Composite International Diagnostic Interview (CIDI-K). Both these instruments have been previously validated and used for large-scale mental health survey studies in Sri Lanka.^15^ ^22^ ^23^ An ‘any CMD’ variable was created to group those who were positive for any one of the constituent disorders (depression, anxiety, somatoform disorder and PTSD) for analytical purposes.^15^

Social support was measured using the Multidimensional Support Scale (MDSS), an instrument capturing the availability and perceived adequacy of social support in populations facing stressful or challenging situations and longitudinal changes.^24^ ^25^ The MDSS assesses emotional, practical and informational support from three main support groups: close confidants, peers and experts (professionals). The MDSS comprises of 6 subscales with each scored on a Likert scale, generating total scores on its two main outcome components: (1) availability (range 4–48) based on the frequency of supportive behaviour and (2) perceived adequacy (range 3–32) based on the satisfaction expressed by participants.^25^ As a cut-point is not standard on this measure, the total scores were divided by tertiles to create low, medium and high groups of social support availability and perceived adequacy. Internal consistency of the English version has been recorded to be high (Cronbach α >0.75)^25^ and the Cronbach α for the Tamil language version used in COMRAID-R was 0.87. An abbreviated version of Lubben Social Network Scale (LSNS-6) was used to measure social networks.^26^ ^27^ This identifies persons at risk for social isolation defined by a cut-off point of 12 or below from a total score ranging from 0 to 30. The scores are derived from six questions evaluating social ties between the participants and kin (family/relatives) and non-kin (friends). Internal consistency via Cronbach α figures are 0.83 for the original English version^27^ and 0.73 for the Tamil version used in COMRAID-R. Neither MDSS nor LSNS-6 had been previously used in Sri Lanka. They were adapted for the local context during the baseline phase. Both these instruments have been used in diverse settings to look at associations between social support/networks and health outcomes.^25–27^

Using an identical structured questionnaire, demographic and economic indicator information was gathered from the participants for age (current and at-displacement), marital status, education, employment, current debt and food insecurity at both study phases.^15^

### Statistical analysis

Data analysis was conducted using STATA V.11.^28^ The data set was adjusted and weighted for clustering arising from the sampling design, taking into account camp and household population variations. Demographic and economic indicators as well as CMD prevalence were described using baseline numbers as a comparison. Mean resilience scores were compared between categories, as well as with mental health, social support and social network measures at both time points, using unadjusted linear regression models to quantify associations. On a matrix constructed for all variables used in the analysis, collinearity was observed between employment and gender (r=0.75; p<0.001), marital status and age at displacement (r=−0.36; p<0.001), education and age at displacement (r=−0.38; p<0.001), and food security and perceived availability of social support (r=−0.43; p<0.001). Multivariable linear regression analyses were carried out to compare resilience score distributions by demographic, economic and social support/network factors at both baseline and follow-up time points. In the final step, multivariable linear regression analyses explored associations between mental health outcomes and resilience, adjusted for demographic, economic and social variables through several models. Model 1 included resilience and any CMD. Model 2 was adjusted for demographic factors of displacement age, gender, marital status and education. Model 3 was adjusted for economic factors of employment, financial debt and food insecurity. Model 4 was adjusted for social support and social networks. Model 5 included variables from models 3 and 4, and the final, model 6, included all the variables from models 4 and 5. Finally, baseline resilience was compared in linear regression models between follow-up groups with/without incident CMD (ie, of those with no CMD at baseline) and between people with/without maintenance of CMD (of those with CMD at baseline).

## Results

### Resilience scores and changes

Of a total of 450 participants recruited at baseline (mean age: 37.1 years; SD: 12.2), 338 (75.1%; mean age at baseline: 38.5; SD: 11.9) were traced and re-interviewed, and 112 (24.9%) were lost to follow-up (mean age at baseline: 36.0; SD: 12.8). The mean resilience score of the baseline sample was 80.2 (95% CI 78.6 to 81.9), mean score of the follow-up sample was 84.9 (95% CI 83.5 to 86.3) and the correlation was negative (r=−0.0107; p=0.844). Mean of the difference between individual resilience score changes at baseline and follow-up was 17.1 (SE: 0.78). The mean resilience score at baseline in those lost to follow-up was 76.2 (73.1 to 79.3). Associations between baseline characteristics and loss to follow-up are described in table 1. Loss to follow-up was more common in those who were never married, in employment and without a mental disorder at baseline. Although loss to follow-up was lowest in those with highest resilience at baseline, there was no clear trend across resilience levels or levels of social support.

**Table 1:** Description of the lost to follow-up group

Baseline characteristic	Number at baseline	N (%) loss to follow-up	Attrition OR (95% CI)
Total sample	450	112 (24.9)	–
Age			
18–21 (born in displacement)	39	9 (23.1)	Reference
22–37 (child at displacement)	189	66 (34.9)	0.8 (0.4 to 1.9)
38–65 (adult at displacement)	222	37 (16.7)	0. 6 (0.2 to 1.3)
Gender			
Male	166	50 (30.1)	Reference
Female	284	62 (21.8)	0.6 (0.4 to 1.0)
Marital status			
Married	345	79 (22.9)	Reference
Widowed/divorced	37	7 (18.9)	0.8 (0.3 to 1.8)
Never married	67	26 (38.9)	**2.1 (1.2** to **3.7)**
Education			
Postsecondary (AL)	61	18 (29.5)	Reference
Secondary (OL)	272	66 (24.3)	0.8 (0.4 to 1.4)
Primary (grade 5)	115	28 (24.3)	0.8 (0.4 to 1.5)
Employment			
Employed	182	58 (31.9)	Reference
Unemployed	268	54 (20.1)	**0.5 (0.3** to **0.8)**
Financial debt			
No debts	256	58 (22.6)	Reference
Indebted	193	53 (27.5)	1.3 (0.8 to 1.9)
Food security (year)			
Sufficient food	319	76 (23.8)	Reference
Lack sufficient food	130	36 (27.7)	1.2 (0.8 to 1.9)
Mental disorder			
Any CMD negative	365	98 (26.8)	Reference
Any CMD positive	85	14 (16.5)	0.5 (0.3 to 1.0)
Somatoform negative	387	103 (26.6)	Reference
Somatoform positive	63	9 (14.3)	**0.4 (0.2** to **0.9)**
Major depression negative	425	110 (25.8)	Reference
Major depression positive	24	2 (8.3)	0.3 (0.1 to 1.1)
Other depression negative	404	102 (25.2)	Reference
Other depression positive	45	10 (22.2)	0.8 (0.4 to 1.8)
Anxiety negative	413	108 (26.1)	Reference
Anxiety positive	30	3 (10.0)	0.3 (0.1 to 1.1)
PTSD negative	439	110 (25.1)	Reference
PTSD positive	11	2 (18.2)	0.7 (0.1 to 3.1)
Resilience			
Very low	49	13 (26.5)	**2.1 (1.0** to **4.6)**
Low	43	13 (30.2)	**2.6 (1.2** to **5.5)**
On low end	55	19 (34.5)	**3.1 (1.6** to **6.2)**
Moderate	63	19 (30.1)	**2.5 (1.3** to **5.0)**
Moderately high	60	22 (36.7)	**3.4 (1.7** to **6.6)**
High	180	26 (14.4)	Reference
Social support			
Availability low	75	17 (22.7)	1.5 (0.8 to 2.9)
Availability medium	175	63 (36.0)	**2.9 (1.8** to **4.8)**
Availability high	200	32 (16.0)	Reference
Adequacy low	76	23 (30.2)	1.6 (0.9 to 2.8)
Adequacy medium	133	37 (27.8)	1.4 (0.9 to 2.3)
Adequacy high	241	52 (21.6)	Reference
Social network			
Adequate network	376	96 (25.5)	Reference
Social isolation	74	16 (21.6)	1.2 (0.7 to 2.7)

Bold values are significant at p<0.001.

AL, advanced level; CMD, common mental disorders; OL, ordinary level; PTSD, post-traumatic stress disorder.

### Associations of resilience with sociodemographic factors and mental health

Distributions of mean resilience scores at baseline and follow-up in relation to covariates are displayed in table 2. At both time points, and in adjusted analyses, lower resilience was strongly associated with food insecurity, lower availability of social support and social isolation. Associations with other covariates were less consistent. Associations found at baseline between lower resilience, male gender and unemployment were not present at follow-up, whereas indebtedness was associated with lower resilience at follow-up but not at baseline. An association between widowed or divorced status and lower resilience was significant at follow-up but not at baseline; however, coefficients were similar in strength and direction at both times.

**Table 2:** Distributions of resilience scores and associations with demographic, economic and social factors

Characteristic*	Baseline COMRAID 2011			COMRAID-R follow-up 2012		
	Mean (95% CI) resilience score	Unadjusted B-coefficient (95% CI)	Adjusted B-coefficient (95% CI)†	Mean (95% CI) resilience score	Unadjusted B-coefficient (95% CI)	Adjusted B-coefficient (95% CI)†
Total sample	80.24 (78.64 to 81.85)	–	–	84.89 (83.45 to 86.34)	–	–
Age						
18–21	85.55 (81.59 to 89.51)	Reference	Reference	85.50 (79.90 to 91.09)	Reference	Reference
22–37	81.66 (79.61 to 83.72)	−3.88 (−10.53 to 2.76)	0.31 (−6.55 to 7.19)	85.90 (83.92 to 87.87)	−0.03 (−6.48 to 6.41)	−1.80 (−7.75 to 4.14)
38–65	77.55 (74.76 to 80.35)	**−7.99 (−14.74** to **−1.24)**	−0.93 (−8.46 to 6.59)	84.00 (81.73 to 86.27)	−1.92 (−8.40 to 4.55)	−1.72 (−7.99 to 4.55)
Gender						
Male	79.03 (76.39 to 81.66)	Reference	Reference	86.29 (83.88 to 88.69)	Reference	Reference
Female	80.95 (78.93 to 82.97)	1.92 (−1.39 to 5.25)	**6.30 (1.65** to **10.95)**	84.28 (82.47 to 86.10)	−1.54 (−4.60 to 1.50)	0.64 (−3.19 to 4.48)
Marital status						
Married	80.37 (78.58 to 82.17)	Reference	Reference	86.10 (84.61 to 87.58)	Reference	Reference
Widowed/divorced	72.56 (64.74 to 80.38)	**−7.81 (−13.62** to **−1.99)**	−4.46 (−10.07 to 1.14)	74.87 (68.95 to 80.79)	**−10.88 (−15.80** to **−5.97)**	**−5.13 (−9.87** to **−0.39)**
Never married	84.28 (81.20 to 87.35)	3.90 (−0.58 to 8.39)	2.65 (−2.26 to 7.57)	85.59 (80.30 to 90.88)	−1.13 (−6.46 to 4.18)	−4.46 (−9.63 to 0.70)
Education						
Postsecondary (AL)	85.78 (82.18 to 89.38)	Reference	Reference	87.27 (83.51 to 91.02)	Reference	Reference
Secondary (OL)	81.25 (79.30 to 83.20)	−4.53 (−9.28 to 0.21)	−0.56 (−5.08 to 3.96)	86.11 (84.43 to 87.78)	−1.54 (−5.67 to 2.58)	0.47 (−3.03 to 3.97)
Primary (grade 5)	75.02 (71.40 to 78.64)	**−10.76 (−16.06** to **−5.45)**	−4.32 (−9.79 to 1.14)	80.36 (76.80 to 83.91)	**−6.97 (−11.76** to **−2.19)**	−2.30 (−6.71 to 2.10)
Employment						
Employed	81.29 (78.91 to 83.67)	Reference	Reference	86.22 (83.95 to 88.48)	Reference	Reference
Unemployed	79.53 (77.38 to 81.69)	−1.75 (−5.02 to 1.51)	**−5.65 (−10.23** to **−1.06)**	84.33 (82.44 to 86.21)	1.88 (−1.08 to 4.85)	3.38 (−0.28 to 7.05)
Financial debt						
No debts	81.09 (78.97 to 83.20)	Reference	Reference	88.04 (86.55 to 89.52)	Reference	Reference
Indebted	79.04 (76.57 to 81.51)	−2.04 (−5.29 to 1.19)	1.11 (−1.90 to 4.12)	76.74 (73.75 to 79.73)	**−11.63 (−14.72** to **−8.53)**	**−4.60 (−7.94** to **−1.26)**
Food security (year)						
Sufficient food	84.73 (83.13 to 86.34)	Reference	Reference	88.62 (87.28 to 89.96)	Reference	Reference
Lack sufficient food	69.09 (65.90 to 72.28)	**−15.64 (−18.87** to **−12.40)**	**−14.93 (−18.22** to **−11.64)**	70.48 (67.50 to 73.46)	**−17.73 (−20.81** to **−14.65)**	**−14.57 (−18.24** to **−10.90)**
Social support availability						
Low	65.84 (61.97 to 69.70)	**−25.27 (−29.02** to **−21.52)**	**−20.90 (−24.91** to **−16.89)**	67.86 (63.74 to 71.97)	**−23.31 (−27.77** to **−18.84)**	**−16.69 (−21.40** to **−11.7)**
Medium	74.00 (71.64 to 76.35)	**−17.11 (−19.98** to **−14.24)**	**−15.50 (−18.38** to **−12.61)**	79.97 (77.65 to 82.30)	**−11.19 (−13.77** to **−8.62)**	**−7.28 (−9.93** to **−4.64)**
High	91.11 (89.60 to 92.62)	Reference	Reference	91.17 (89.76 to 92.58)	Reference	Reference
Social support adequacy						
Low	80.65 (76.26 to 85.05)	−3.48 (−7.79 to 0.82)	−0.45 (−4.54 to 3.63)	83.56 (78.97 to 88.15)	−0.67 (−5.10 to 3.75)	0.03 (−3.73 to 3.80)
Medium	72.95 (69.91 to 75.99)	**−11.18 (−14.72** to **−7.64)**	**−8.35 (−11.74** to **−.97)**	86.30 (83.97 to 88.62)	2.05 (−1.09 to 5.21)	1.15 (−1.50 to 3.81)
High	84.14 (82.25 to 86.02)	Reference	Reference	84.24 (82.25 to 86.23)	Reference	Reference
Social network						
Adequate network	79.41 (77.64 to 81.18)	Reference	Reference	86.00 (84.49 to 87.50)	Reference	Reference
Social isolation	84.47 (80.75 to 88.19)	**−5.05 (−9.36** to **−0.74)**	**−5.31 (−9.24** to **−1.37)**	78.65 (74.30 to 82.99)	**−6.67 (−10.88** to** −2.46)**	**−6.51 (−10.02** to **−2.99)**

Bold values are significant at p<0.001.

*Measured contemporaneously with resilience at each examination.

†Adjusted for age, gender, marital status, education, employment, debt and food security.

Table 3 presents unadjusted analyses of resilience levels according to CMD and constituent mental disorders. At both time points, there were significant associations with CMD, with comparable strengths of association. ‘Other depression’ appeared to account for most of this association at baseline, with resilience, if anything, higher rather than lower in other constituent disorders, apart from PTSD. At follow-up, the same was true for ‘other depression’, but major depression was also significantly associated with lower resilience, and the coefficient for somatoform disorder was negative rather than positive.

**Table 3:** Distribution of resilience scores by mental disorder and linear regression coefficients

Characteristic	Baseline COMRAID (2011)		COMRAID-R follow-up (2012)	
Mental disorder	Mean (95% CI) resilience score	Unadjusted B-coefficient (95% CI)	Mean (95% CI) resilience score	Unadjusted B-coefficient (95% CI)
No CMD	81.33 (79.75 to 82.91)	Reference	85.41 (83.95 to 86.87)	Reference
Any CMD	75.56 (70.55 to 80.57)	**−5.77 (−9.84** to **−1.70)**	79.67 (73.80 to 85.54)	**−6.19 (−11.41** to **−0.97)**
Somatoform	84.07 (79.49 to 88.66)	4.45 (−0.15 to 9.06)	81.50 (74.80 to 88.19)	−4.02 (−10.30 to 2.26)
Major depression	82.45 (73.12 to 91.78)	2.34 (−4.81 to 9.49)	70.85 (53.79 to 87.92)	**−14.33 (−24.40** to **−4.26)**
Other depression	66.64 (59.83 to 73.45)	**−15.11 (−20.28** to **−9.93)**	74.66 (64.14 to 85.18)	**−11.47 (−20.67** to **−2.27)**
Anxiety	81.26 (73.05 to 89.47)	1.24 (−5.21 to 7.71)	43.00 (0 to 0)	*
PTSD	77.54 (62.10 to 92.98)	−2.76 (−13.17 to 7.63)	61.00 (0.0)	*

Bold values are significant at p<0.001.

*Insufficient cell sizes.

CMD, common mental disorders; PTSD,post-traumatic stress disorder.

Adjusted associations between CMD and resilience are displayed in table 4. At baseline, social measures were negative confounders and the association remained significant after full adjustment. At follow-up, the association was weakened by all individual adjustments and was no longer significant in the final, fully adjusted model.

**Table 4:** Linear regression models of the difference in resilience scores between those with and without any common mental disorder

Model	Baseline COMRAID (2011)		COMRAID-R follow-up (2012)	
	Regression coefficient (95% CI) (p)	Adjusted R^2^	Regression coefficient (95% CI) (p)	Adj. R^2^
Model 1	**−5.77 (−9.84** to **−1.70)** **(p=0.006)**	0.0148	**−6.19 (−11.41** to **−0.97)** **(p=0.020)**	0.0130
Model 2	−3.85 (−8.11 to 0 0.40) (p=0.076)	0.0480	−4.14 (−9.40 to 1.11) (p=0.122)	0.0500
Model 3	−3.60 (−7.44 to 0.23) ( p=0.066)	0.1710	**−4.87 (−9.27** to **−0.46)** **(p=0.030)**	0.3054
Model 4	**−7.86 (−11.03** to **−4.70)** **(p=<0.001)**	0.4224	**−4.93 (−9.13** to **−0.73)** **(p=0.021)**	0.3809
Model 5	−1.53 (−5.54 to 2.47) (p=0.453)	0.1944	−3.89 (−8.37 to −0.58) (p=0.088)	0.3174
Model 6	**−4.91 (−8.28** to **−1.55)** **(p=0.004)**	0.4548	−3.21 (−7.23 to 0.80) (p=0.117)	0.4604

Bold values are significant at p<0.001.

1. Unadjusted (resilience scores modelled against any CMD (binary variable)).

2. Adjusted for displaced age, gender, marital status, education.

3. Adjusted for employment, food security, financial debt.

4. Adjusted for social support, social network.

5. Adjusted for model displaced age, gender, marital status, education, employment, food security, financial debt.

6. Adjusted for model displaced age, gender, marital status, education, employment, food security, financial debt, social support, social network.

At baseline, the prevalence of any common mental disorder was 18.8%. Out of the 85 participants with CMD at baseline, 71 participants (83.5%) were followed-up. The 1-year maintenance of CMD was 28.2% (95% CI 18.6 to 37.7) in those followed-up. In 267 (73%) participants followed from the 365 with no CMD, incidence of CMD was 2.2% (0.7 to 3.7). In an analysis regressing mean baseline resilience scores against CMD maintenance, the unadjusted B-coefficient was −3.33 (−11.64 to 4.97) and that adjusted for age, gender, marital status, education, employment, debt and food security was −1.33 (−9.36 to 6.69). The unadjusted and adjusted B-coefficients against CMD incidence were −0.74 (−12.43 to 10.95) and −1.46 (−12.07 to 9.14), respectively.

## Discussion

### Principal findings

We report one of the first longitudinal studies with a 1 year follow-up of 338 participants from a baseline number of 450 to explore resilience, its dynamics and the link to mental health among a conflict-affected adult population experiencing prolonged displacement in the global context, using a resilience-specific measurement. Our findings were that resilience was most influenced by food security and social support. The association between resilience and mental health was inconsistent in cross-sectional analyses and potentially confounded by demographic, economic and social factors. It was also not predictive of incidence or maintenance of CMD over a 1-year period in this sample.

### Resilience dynamics

Previous research has reported that, in general, resilience has a negative association with prolonged displacement.^29^ ^30^ Our study findings are partly similar, and partly contradictory of this, as the baseline resilience levels were generally lower (potentially associated with prolonged displacement) and follow-up levels were higher (potentially associated with conflict-cessation and prospect of return). However, issues with measurement equivalence and lack of comparable studies prevent a more accurate assessment. It has also been suggested that continuous adversity together with being in a displaced state for long periods has a detrimental effect on forced migrants, especially for older age groups and women,^29^ ^31^ ^32^ although our study found no independent and/or consistent age or gender differences in resilience levels. Adolescent groups of forced migrants have shown increased resilience levels after end of displacement, correlating with increasing time interval,^12^ and a systematic review by Tol *et al*,^10^ (limited to studies on resilience among conflict-affected children and adolescents), concluded that resilience is a process driven by time and context specific factors. A recent systematic review also highlighted the social-ecological nature and the complex individual and communal trajectories of resilience.^33^ The general increase in resilience levels at follow-up for our own sample might reflect the cessation of the conflict that had given rise to the original displacement, the increasing possibility of return migration and the decrease in continuing conflict-related adversity.^15^ ^17^ However, it should be borne in mind that loss to follow-up was higher in those found to have lowest levels of resilience at baseline and differential attrition might therefore have had an influence in the change in levels between examinations.

### Socioeconomic correlates

Widowed and divorced members of this IDP population tended to have lower resilience scores—a difference that was significant at follow-up and comparable in strength at baseline as well. Other adverse experiences such as lower education levels and poverty-related factors (eg, lack of food security and financial debt) are also linked to lower levels of resilience.^9^ ^33^ In our study, financial debt and lack of food security had both shown a substantial negative influence on resilience levels. Although the influence of financial debt on resilience scores was only stronger at follow-up, lack of food security showed a strong negative association with resilience scores at both phases. While these findings indicate that adverse events not directly linked to the displacement ordeal may impact on the individual resilience, possibly compounding the traumatic experience of forced migration, lack of comparative data prevents a better understanding of their role.^33^ However, these factors can be considered as a part of daily stressors experienced by the forced migrants during the postdisplacement period, a combination of which may contribute to the decreasing of a resilience threshold.^34–36^ For example, the influence of debt on resilience levels at the follow-up may be related to the likelihood of return migration closer to the follow-up phase. The findings support the claim that resilience is not only affected by damaging psychological experiences, but also by environmental, economic and cultural adversities.^9^ ^33^ ^37^ Hence, it is important for public health and administrative services to consider addressing issues such as food security when managing displaced populations in the future.^33^ Low availability of social support and social isolation were strongly associated with lower resilience in this sample. Adequately available social support and strong social networks have been previously recognised as contributory factors for improved resilience among displaced populations.^3^ ^32^ ^33^ ^38–40^ Social support and social networks have also been found to play a role in the possibility of developing mental disorders in high-risk populations such as IDP and refugees.^39^ ^41^ However, in our sample, availability of social support and isolation both had stronger relationships with resilience than perceived adequacy of support. Apart from availability and adequacy, social support varies by its nature, function and importance.^42^ These variations may define how support interacts with or impacts on individual functioning, where availability of tangible support (especially in adverse social situations such as displacement) may take precedence over its perceivable adequacy,^42^ potentially explaining the stronger influence of the availability of social support on resilience in our study. However, it must be noted that our observations maybe also be due to context/culture/area specific factors related to social support.

### Mental health and resilience

The relationships between mental health measures and resilience were substantially less consistent than those for other exposures such as food insecurity and social support. Resilience levels related to individual mental disorders varied between the two study phases in ways which would not be accounted for by statistical power (since associations tended to be stronger, if anything, in the smaller follow-up sample). The association between resilience and CMD as a whole was not consistent and demographic, economic and social measures appeared to be important potential confounders. Finally, there was little evidence for resilience levels at baseline predicting either incidence or maintenance of CMD over the follow-up period, although numbers were relatively small, particularly for the analysis of incidence. Differential attrition does not appear to be an obvious reason for this lack of association since those lost to follow-up had lower resilience but better mental health at baseline. This points to the fact that contextual variations in demographic and economic factors over time, especially factors such as employment, debt or food security, may actively impact on levels of resilience and mental health.^9^ ^33^ ^43^ Although the impact of these factors on mental health has been well established,^7^ ^33^ ^43^ evidence related to the impact on resilience, or the mental health-resilience nexus, is limited, especially for adult populations, compounded by the lack of longitudinal studies.^33^ The CMD maintenance rate observed in the follow-up group is also important in interpreting the resilience findings, as the participants were not subjected to any specific psychiatric intervention to our knowledge. However, there is lack of comparable data from other postconflict settings on CMD maintenance, which makes definitive conclusions difficult. Given the resource-poor settings where most conflict-affected populations live, understanding these contextual variations is critically important for effective management of mental healthcare provision, including intervention development.^33^

### Strengths and limitations

Strengths of the study include complete participation at baseline and a reasonable follow-up rate despite the complex postconflict mobility of the population, maximising generalisability and reducing likelihood of bias. Differential attrition does need some consideration, however, as those lost to follow-up differed significantly in several respects from those followed. In general, the loss to follow-up was consistent with a healthy migrant effect, being highest in participants who were never married, who were employed and who did not have a mental disorder at baseline, although, interestingly, it was associated with lower rather than higher resilience at baseline. We believe this study is the first of its kind to measure resilience longitudinally by using a specific tool among a population of forced internal migrants affected by conflict-driven prolonged displacement. It also considers a range of important demographic, economic and social variables consistently across two time points, coinciding with the assumed reduction in adversity in postconflict and return migration periods. It addresses key gaps in the field of resilience research by being longitudinal in design and by exploring an adult population.^9^ ^10^ Limitations include the appropriateness of the resilience measuring instrument and selection of time points. Capturing of only two time points during a 1 year follow-up may have limited the information that would have potentially been captured by the longitudinal approach. Although the RS-14 has been used in different contexts and among different population groups such as children, adolescents, older adults and women, this is the first time to, our knowledge, that it had been used in a conflict-affected adult population.^18^ Two reviews on resilience measuring instruments found the RS to have the best psychometric properties among many available instruments.^44^ ^45^ The selection of RS-14 for this study was based on its wider usage, relatively higher validity and the fact that it has shown previous associations with mental health, trauma and functioning outcomes in different settings.^18^ ^44^ ^45^ We cannot be certain that the RS-14 actually captured the concept of resilience as broadly defined in the introduction due to the complex nature of the construct itself and cultural/contextual variations of resilience. In addition, cultural validity of the instrument may also have affected the results.^37^ Test–retest or inter-rater reliability testing were not carried out for the measures used in the study including RS-14, MDSS and LSNS-6, which is another potential limitation. However, adequate internal consistency of the translations and a strong solution in the factor analysis support the robustness of the RS-14 in this context. Lastly, sample size and statistical power limitations may have affected the incidence and maintenance-related analyses, and analyses related to CMD subgroups. The number of participants studied in the follow-up (in determining CMD incidence) may have led to reduced statistical power as opposed to a sample size calculated specifically for determining the CMD incidence. Missing data were handled using a combination of complete case analysis and last observation carried forward methods.

Our findings point towards the need for more longitudinal studies of resilience as a construct that interacts with mental health among traumatised populations.^33^ Such studies stand to provide a more coherent understanding of the temporal interactions between resilience and mental health, and its protective role in preventing psychopathology. The study also highlights the need to integrate the resilience research with developing more evidence-based interventions that target individuals as well as communities, and the promotion of resilience-based approach to enhancing mental health at primary care level.^9^ ^33^ ^46^ More research exploring the dynamics of resilience is needed to enhance the emerging primary evidence base for intervention development.^33^ Conflict-driven forced migrant populations, especially those in prolonged displacement, stand to benefit through such research.
